# Comparison of Uterine manipulators types in total Laparoscopic Hysterectomy: A retrospective study

**DOI:** 10.12669/pjms.39.4.6728

**Published:** 2023

**Authors:** Mustafa Sengul, Halime Sen Selim

**Affiliations:** 1 Mustafa Sengul Associated Professor, Obstetrics and Gynecology Department, Izmir Katip Celebi University, Basın Sitesi Mah, Karabaglar/Izmir; 2 Halime Sen Selim Specialist MD, Obstetrics and Gynecology Department, Izmir Katip Celebi University, Ataturk Training and Research Hospital, Basın Sitesi Mah, Karabaglar/Izmir

**Keywords:** Hysterectomy, Laparoscopic hysterectomy, Uterine manipulators

## Abstract

**Objective:**

To compare the results of operations of commercial uterine manipulators. Considering that the optimal uterine manipulator is still not found, our goal was to give an idea for gynecologists to choose the most suitable uterine manipulator for their purposes and expectations.

**Methods:**

Between January 2016 and September 2021, 294 laparoscopic hysterectomy cases met the inclusion criteria and were operated in İzmir Katip Çelebi University Atatürk Training and Research Hospital Gynecology and Obstetrics Clinic. They were divided into four groups according to the type of manipulator used. Group-1 (RUMI-I), Group-2 (Clermont-Ferrand), Group-3 (sharp intrauterine curette-tenaculum), and Group-4(without uterine manipulator). All four groups were compared with operation time, hospital stay, absolute change in hemoglobin (g/dl), and per-op complications.

**Results:**

Considering the operation times in all groups, the mean operation time in the first Group-was 180 minutes (98-349); in Group-2 was 159 minutes (96-564); in the 3rd Group was 178 minutes (141-540); in the 4th Group was 189 minutes (115-453). The group with the shortest operation time was Group-2; the difference was statistically significant (p=0.015). In general terms, the effect of all manipulators on patient parameters wasn’t very different. Clermont-Ferrand seems more advantageous than others in making the surgeon’s work more straightforward regarding operation time only.

**Conclusions:**

In our study, the choice of uterine manipulator did not affect the surgical results except for the duration of the operation. The personalization of uterine manipulators according to the needs of the surgery and the easiness of use of the surgeon should be at the forefront.

## INTRODUCTION

Hysterectomy is one of the most common gynecological surgical procedures performed abdominal, vaginal, and laparoscopic.^1^ In the 19th century, the first vaginal or abdominal hysterectomy was successfully performed.^2^ Laparoscopic hysterectomy (LH), which has started to be preferred more frequently in recent years with the rapid development of technology, was first performed in 1989.^3^ Due to its advantages like less pain in the postoperative period, shorter recovery time, early discharge, minimal wound infection risk, faster return to work, cosmetic, total laparoscopic hysterectomy has become an indispensable surgical option for gynecologists, which has been increasing in recent years.^4^

In laparoscopic hysterectomy cases, which have become more preferred with the rapid development of technology in recent years, in addition to the surgeon’s experience, the characteristics of the instruments and devices used have gained importance, and various companies have produced different products. The ideal uterus manipulator is cheap and convenient, eliminating the need for an assistant and offering the surgeon a good range of motion.^5^ Uterine manipulators facilitate dissection of the uterine artery, reduce cases of endometriosis, help to clearly define the vaginal fornix at the colpotomy stage, prevent pneumoperitoneum after vaginal incision, and increase the distance between the cervix and ureter thanks to the intraabdominal traction of the uterus, thus allowing safer dissection around the cervix. In particular, they reduce the risk of ureteral injury. Because of this, these devices have become indispensable.^6^,^7^

Clermont-Ferrand (CF) uterine manipulator, handle with fixing screw; manipulator bar with five locking positions between 0° and 90°; silicone sealant; it is a reusable tool consisting of a rotating ceramic head.^8^ CF uterine manipulator provides 140° movement of the uterus in the anterior and posterior directions and manipulation in the lateral plane. It also has the feature of stretching the uterus on itself. Its stepped Snap-on mechanism with five different positions provides stability to the uterus at various angles. When the manipulator rod is pushed forward, it helps define the vaginal fornix with the help of the porcelain cap attached to the front. It has a series of silicone gaskets to prevent pneumoperitoneum after the colpotomy incision. Significant disadvantages are that it requires cervical dilatation up to size nine before insertion into the cervix and requires a great deal of training to use the device correctly.^9^

The RUMI system consists of Koh cervical cap and Koh colpo-pneumo-occlusive. RUMI manipulator is a versatile uterine manipulator with perfect uterine manipulation in the anterior, posterior and lateral planes. It also helps in the straightforward identification of the vaginal fornix. It helps to complete laparoscopic dissection of the vagina much more easily, providing greater efficiency and less blood loss while eliminating difficulties with vaginal access. This enhanced uterine mobility also accelerates utero-vesical peritoneal dissection and downward displacement of the bladder.^10^ We aimed to investigate whether different uterine manipulators affect patient parameters (operation time, length of hospital stay, absolute change in hemoglobin,per-op complications) in our hospital’s LH operations performed with benign indications.

## METHODS

Patients who were operated at Izmir Katip Çelebi University Atatürk Training and Research Hospital Gynecology and Obstetrics Clinic between January 2016 and September 2021 were included in the study. A total of 356 patients underwent laparoscopic hysterectomy operations. This study was conducted with 294 who met the inclusion criteria. Written informed consent was obtained from all patients. Information about the patients was obtained from the records of İzmir Katip Çelebi University Atatürk Training and Research Hospital and analyzed retrospectively. Ethics committee approval was received for this study from Izmir Katip Çelebi University Medical Ethics Committee (approval date 23.12.2021, approval reference number 544).

Bimanual pelvic examination, a transvaginal ultrasound, examination with cervicovaginal smear, and endometrial sampling were performed before the operation. Patients with suspected malignancy as a result of smear and endometrial sampling were excluded from this study. Six cases due to malignancy were excluded from the study.

Those who underwent TLH(n=36), TLH+bilateral salpingo-oophorectomy (n=294) and TLH+bilateral salpingo-oophorectomy + Additional procedures (Colporaphy, Transobturator Tape, Perineoplasty, etc.) (n=20) for benign reasons were evaluated. Cases, where an additional procedure was applied, were excluded from the study because it may affect the operation time, hospital stay, and hemoglobin changes.

Two hundred ninety-four LH cases were analyzed in terms of age and operation indications retrospectively and divided into four groups according to the preferred manipulator type; Group-1 (RUMI-I Cooper Surgical, Trumbull, CT, USA) (n=124), Group-2 (Clermont-Ferrand Karl StorzGmbhandCo, Tuttlingen, Germany) (n=70) and Group-3 (sharp intrauterine. Curette-tenaculum) without commercial uterine manipulator (n=54) and Group-4 (without uterine manipulator) (n=46). The same surgical team performed all operations. In the third group, a commercial uterine manipulator hasn’t used; uterine manipulation was achieved with a Tenaculum -which is a single tooth forceps- at the 11 o’clock point of the cervix and inserting a number five sharp curette into the uterus after cervical dilatation, and fixing the handle to the tenaculum with the help of gauze. In the fourth group, we didn’t use any type of uterine manipulator.

All four groups were evaluated in terms of age and operation indications. Also, they were compared with parameters such as operation time, hospital stay (days), Pre-op hemoglobin-post-op hemoglobin values, absolute change in hemoglobin [ difference between the preoperative hemoglobin (Hb) (g/dl) value and the postoperative Hb(g/dl) value], per-op complications (transition to laparotomy, ureter injury, bladder injury, vaginal cuff separation, vaginal laceration, vaginal hematoma), uterine length in the pathology specimen.

**Fig.1:**
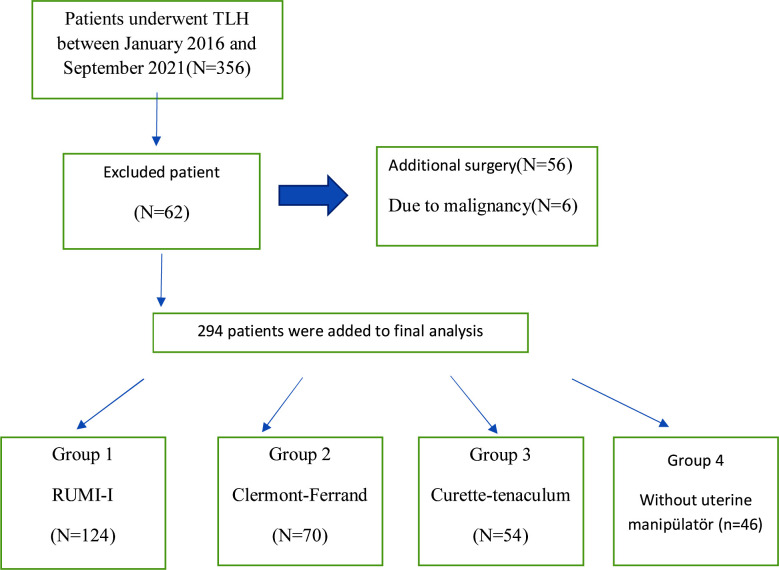
Final analysis.

### Data collection:

Data including preoperative evaluations, imaging, surgery notes, and pathology reports were obtained from patient management system records. Operation times were described as minutes per the information in the anesthesia follow-up forms. The uterine length was determined as centimeters, according to histopathological examination results.

### Surgery Technique:

UM was placed in all patients in the dorsal lithotomy position under general anesthesia by the surgeon. The abdomen was infused with a Veress needle. A 10 mm trocar was inserted through the umbilicus. Two 5mm trocars were placed on both sides, 1/3 lateral of the imaginary line between the right and left anterior iliac superior (SIAS) and the umbilicus. A 5mm trocar was placed at the midpoint of both SIAS. The infundibulopelvic ligaments were coagulated, sealed, and cut. The uterovesical fold was dissected from the anterior vaginal wall. Uterine arteries were coagulated and cut bilaterally. Ligasure (Covidien, USA) was used for coagulation and cutting. Colpotomy was performed with a monopolar L-hook cautery device or a harmonic scalpel (Ethicon, Endo-surgery, USA). The vagina was sutured vaginally with 1.0 Vicryl using a simple intermittent suture technique.

### Statistical analysis:

Statistical analysis was performed using Statistical Package for the Social Sciences (SPSS) software (SPSS, Version 22; SPSS Inc, Chicago, IL). Descriptive variables were presented as median, minimum, and maximum values. The Kruskal-Wallis test compared the means of more than two independent groups in continuous variables determined by measurement, and the Bunn-Bonferroni test for post hoc pairwise comparisons. Pearson Chi-square test was used for categorical variables. The significance level was accepted as 0.05.

## RESULTS

The mean age of the four groups was similar. The mean age among the groups, respectively; was 48 years; 48 years; 52 years; 48 years (p=0.616). Also, pre-op hemoglobin values of all groups were similar in all groups, respectively, 12.3 g/dl; 12.6 g/dl; 12.8 g/dl; 12.6 g/dl (p=0.732).

Median values of uterus lengths measured in pathology specimen were 9 cm (5.5-16); 9cm(5,-14); 9cm(4-15); 9.5 cm (4-12), and there was no statistical difference between the groups (p=0.837). The general characteristics of the patients between groups are summarized in Table-I.

**Table-I:** General characteristics of the patients

	Group-1 Mean (minimum-maximum)	Group-2 Mean (minimum-maximum)	Group-3 Mean (minimum-maximum)	Group-4 Mean (minimum-maximum)	P-value
Age (year)	48 (21-74)	48 (38-71)	52 (41-69)	48 (39-66)	0.616
Preoperative hemoglobin(g/dl)	12.3 (8.2-15.5)	12.6 (8.9-16.2)	12.8(8.2-14.9)	12.6 (9.6-15.8)	0.732
Postoperative hemoglobin(g/dl)	10.5 (8.1-11.1)	10.7 (8.7-10.8)	11.4(8.0-11.9)	10.1 (9.5-11.4)	0.686
Uterine lengths(cm)	9cm(5.5-16)	9cm (5-14)	9cm (4-15)	9.5cm (4-12)	0.837

The operation indications of our patients are similar, and the distribution of operation indications is summarized in Table II.

**Table-II:** The distribution of operational indications.

Operational indications	Group-1 (n=124; %100)	Group-2 (n=70; %100)	Group-3 (n=54; %100)	Group-4 (n=46; %100)
Myoma uteri	36(%29)	24 (%34,3)	30 (%55,6)	22((%47,8)
HSIL	6 (%4,8)	4 (%5,7)	0(%0)	2 (%4,3)
Adnexal mass	30 (%24,2)	12 (%17,1)	12(%22,2)	4(%8,7)
Treatment-resistant menometrorrhagia	20 (%16,1)	14 (%20)	2(%3,7)	0 (%0)
Endometrial hyperplasia	16 (%12,9)	12 (%17,1)	2(%3,7)	10 (%21,7)
Prolapsus	6 (%4,8)	0 (%0)	2(%3,7)	4(%8,7)
Other	10 (%8,1)	4 (%5,7)	6(%11,1)	4(%8,7)

HSIL: high-grade squamous intraepithelial lesion.

Considering the operation times in all groups, the 1st Group was 180 minutes (98-349). Group-2 159 minutes (96-564); 3rd Group 178 min (141-540); Fourth Group-189 (115-453) minutes; the group with the shortest operation time was the CF Group; the difference was statistically significant (P=0.015).

When we look at the pre-op/ post-op hemoglobin change to evaluate absolute blood loss more accurately, although blood loss seems to be higher in the 2nd group, no statistically significant difference was observed (1st Group hemoglobin average 1.8 (0.1-4,4 g/dl); 2. Group 1.9 (0.2-5.4) g/dl; 3.Group 1.4 (0.2-4.3) g/dl; 4. Group 1.5 (0.1-4.5) g/dl (P=0.094). The length of hospital stay time were similar in all groups; the average was two days in all groups (p=0.282).

When the development of per-op complications in all groups was also examined, 6.5% (n=8) in Group-1; 8.6% (n=6) in Group-2; 0% in Group-3 (n=0); 13% (n=6) in Group-4 (p=0.281) but It was observed that there was no statistically significant difference. The comparison of surgical outcomes (operation time, absolute change in hemoglobin, length of hospital stay, per-op complications )of the patients between groups is summarized in Table- III.

**Table-III:** Comparison of surgical outcomes of four groups.

	Group-1 (*n*=124; %100)	Group-2 (*n*=70; %100)	Group-3 (*n*=54; %100)	Group-4 (*n*=46; %100)	P-value
Operation time (min)^α^	180 (98-349))	159 (96-564);	178 (141-540);	189 (115-453)	0.015*
Absolute change in hemoglobin (g/dl)^α^	1.8 (0.1-4,4)	1.9 (0.2-5.4)	1.4 (0.2-4.3)	1.5 (0.1-4.5)	0.094
Length of hospital stay (days)^α^	2 (1-4)	2 (2-5)	2 (1-6)	2 (1-5)	0.282
Per-op complications	8 (6.5%)	6 (8.6%)	0	6 (13%)	0.281

α Mean ;minimum–maximum

* P<0.05 was considered statistically significant.

## DISCUSSION

This study shows that the operation time is shorter for the CF uterine manipulator in patients undergoing LH. There are many studies on the use of manipulators in laparoscopic hysterectomy. There are few data in the literature comparing different types of uterine manipulators designed to facilitate TLH in terms of their clinical benefits, efficacy, and safety. However, according to our search on PubMed and Scholar Google, no study compares the use of Clermont-Ferrand (CF) and RUMI manipulators. It is perhaps the first study. Although it was not statistically significant in our study, the operation time was longer in the RUMI manipulator group. In a comparative study of the RUMI manipulator with the V Care® manipulator, the operation time (196.4±30.5 min. in RUMI vs. 147.3±38.9min. in V Care®, p =0 .006) was significantly higher in the RUMI manipulator group.^11^ In another study comparing CF with another manipulator (Vectec), CF seems to be disadvantageous in operation time (89 ± 17 min. in CF vs. 81 ± 15 min. in VT, p =0 .004).^12^

In the study of Aslan et al., which compared three manipulators, CF, RUMI, and V Care®, in terms of operation times, no statistically significant difference was found (90 (50–180) minutes. in CF vs. 90 (50–180) minutes. in RUMI vs. 80 (50–130) minutes. in V Care®, p =0 .51).^13^

In the study of Yavuzcan et al.^11^, the operation times of the group using RUMI manipulator in LH were similar to our study [respectively 196.4±30.5 min. vs 180 (98-349 minutes.]. These times are longer than the operation time stated as 90minutes (50–180) in the study of Aslan et al., who used the RUMI manipulator. The reason for this situation is that Aslan et al. defined the duration of time as the time from skin incision to uterine detachment. The time of insertion of the uterine manipulator is also not included in this process.

From this point of view, although the surgeon’s experience is an essential parameter during the operation, the CF manipulator seems to make the surgeon’s work more straightforward than the RUMI manipulator.

On the other hand, although absolute blood loss seems to be a little more in the CF group, statistically significant results are needed to reach a definitive interpretation. Similar to our study, in a survey that compared three manipulators, Clermont-Ferrand, VCare, and RUMI, the group with the highest blood loss was CF.^13^

The lowest absolute hemoglobin change among the groups was 1.4 g/dl (0.2-4.3) in the Curette-buffer group. Similarly, in the report of Dimitrios et al., in 1023 cases where a uterine manipulator was not used, the estimated blood loss was as low as 59 mL (20-260 mL).^14^ In terms of the amount of bleeding, it is seen that using a commercial uterine manipulator does not provide any additional advantage. In the report of Dimitrios et al. on 1023 cases where a uterine manipulator was not used, the mean operation time was 78 minutes (43-168 minutes), the estimated blood loss was 59 mL (20-260 mL), and the mean uterine weight was 255 g (40-1510 g). There was no case of conversion to laparotomy. The mean length of hospital stay was 1.1 days, with only 38 patients staying for two or more days, and the difference between the groups was not statistically significant (P=0.82). Rapid postoperative recovery and short hospital stay appear to be an advantage for laparoscopic hysterectomy patients.^14^ Studies examining length of stay in hospital vary widely. Consistent with our study, Mitri et al. found the length of hospital stays as one day in laparoscopic hysterectomies performed for benign indications.^15^ Kang et al., in their research with the RUMI manipulator, found the average hospital stay to be 4.1 days, unlike our study. It is thought that this is due to the presence of premalignant and malignant patients in the patient group.^16^

Five cases (0.5%) had vaginal cuff detachment in the long term, and one patient had vaginal cuff hematoma.^17^ Beyan et al. evaluated the Uterine manipulator requirement in laparoscopic surgery of Ectopic Pregnancy. They revealed that the operation time was significantly shorter (p<0.001) in the group where uterine manipulators were not used than in the uterine manipulator group.^18^

It is already known that; laparoscopic hysterectomy is more advantageous for postoperative infection ratio and analgesia needs.^19^,^20^ However, different type of uterine manipulator and how to contribute to this is unclear. No complications were observed in the curette-buffer group. However, two cases (0.01%) requiring bladder repair were used in the RUMI manipulator, and one patient (0.56 %) was in the CF group. In our study, the need to laparotomy in six patients (0.5%), bladder injury in three patients, vaginal cuff separation in five patients, vaginal laceration in three patients, and vaginal hematoma in three patients were observed as per-op complications. Vaginal laceration and vaginal hematoma were detected in an equal number of patients (n=3) in the RUMI and CF manipulator groups.

On the other hand, there was no ureter injury in all groups of our study. When we look at the literature, the ureteral injury incidence reported in patients who underwent total laparoscopic hysterectomy is 0.5-1%.^21^,^22^ Kavallaris et al. said that ureteric and bladder injury incidence was 0.5-1% if the uterine manipulator was used; no complication was defined without the uterine manipulator group.^23^ In the report of Dimitrios et al., in 1023 cases where a uterine manipulator was not used, there was a case of ureter injury in one case and a case in which the bladder was opened and fixed laparoscopic ally in three patients.^17^ The limited number of complicated issues is insufficient to reach a definitive conclusion. As a result, in general terms, the effects of both manipulators on patient parameters are not significantly different (P=0.281).

### Limitations of the study:

There are some limitations because our study’s data was obtained retrospectively; like operation, time is include uterine manipulator insertion period and anesthesia premedication period. On the other hand, uterine manipulator insertion time duration can also be a negative parameter for choosing a uterine manipulator.

## CONCLUSION

In our study, the choice of uterine manipulator did not affect the surgical results except for the duration of the operation. Clermont-Ferrand (CF) seems to be more advantageous compared to RUMI and the Curette-buffer group in terms of facilitating the surgeon’s work only in terms of operation time. On the other hand, although it is not statistically significant, the least blood loss group is the curette-buffer group. Commercial uterine manipulators do not seem to contribute much to patient morbidity. However, a statistically significant difference can be found in large patient series. As a result, the personalization of uterine manipulators according to the needs of the surgery and the easiness of use of the surgeon should be at the forefront.

### Author’s Contributions:

**MS**: conception and design, acquisition of data, analysis, and interpretation of data; drafting the article, final approval of the version to be published.

**HSS**: Contributions to conception and design, data acquisition, analysis and interpretation of data, drafting the article or revising it critically for important intellectual content, and final approval of the version to be published.
